# Clinical Report of Three Cases Treated by R. B. Johnstone, M. D.

**Published:** 1888-12

**Authors:** 


### CLINICAL REPORT OF THREE CASES TREATED BY R. B. JOHNSTONE, M. D.,

APPLICANT FOR MEMBERSHIP IN INTERNATIONAL HAHNEMANNIAN ASSOCIATION.

PHILADELPHIA, October 1st, 1887.

TO THE HONORABLE PRESIDENT, BOARD OF CENSORS, AND GENTLEMEN OF THE INTERNATIONAL HAHNEMANNIAN ASSOCIATION:—Having made application for membership in your honorable body, and in accord with the Constitution and By-Laws of your Association, I beg to submit the following report of cases treated by me according to the law of Similia, using the potentized remedy, and the minimum dose. I mention the following cases because they are illustrative of the golden rules which should bind us in closer brotherhood, as well as control our method of practice, that our success in relieving and curing the many agonizing troubles we meet with daily, will be beyond even our own anticipation, which if treated otherwise would unquestionably doom the patient to a life of misery, if not followed by sudden death.

Case I.—Mr. T. J., residing at W., age, forty-one; weight, two hundred and thirty-five pounds; height, five feet and six inches.

August 17th.—About nine weeks ago, noticed an eruption upon the back of the wrists which soon spread so as to encircle the whole of the forearm, next making its appearance about the neck and forehead, the rims of the ears, bridge of nose, and upon the ankles. During this time he had consulted one of the hyphenated degree graduates of a so-called homœopathic college, and received some kind of bitter pill which was supposed to be able to turn the liver around, and cure the disease by righting the refractory organ and cause a cure by heroically striking at the supposed seat of the affection, as an adjuvant to this treatment. He also received an ointment composed of Cosmoline and the Oxide of Mercury, to be used every night, well rubbed in. After two weeks of this treatment, he was decidedly no better, stiffness of the joints in various parts of the body began to appear, the eruption became intensified and extended itself to other parts of the body, most notably the sexual organs: the scrotum became thickened and exceedingly painful, the cellular tissue beneath the skin of scrotum became enormously œdematous, the urine became blood-red, incontinent, alternately profuse and scanty, the glans and prepuce became swollen, dark-red, and erysipelatous. At this juncture of the case he became much alarmed, and consulted another physician of the rational scientific school, who prescribed a solution of Acetate of Lead to be used as a lotion, and Iodide of Potassium internally to antidote the mercurial impression which had been made by the previous prescription. He continued this treatment for two or three weeks without any noticeable change for the better; in fact, the skin symptoms were much intensified, and many other internal symptoms were added. Our patient, now having lost all confidence in doctors and medicine, did nothing but scratch himself for two weeks, when he fell into my hands. During the time which he had suspended medication most of his stomach and intestinal symptoms disappeared. On his first visit to me, I found the following well-defined indications.

October 28th.—Giddy when rising from a recumbent position, with chilliness and pressure in the head, forward. Brain feels as though wabbling about when shaking the head. Hair so sensitive that he can scarcely bear to comb it. Eyelids dark, red, swollen, agglutinated, œdematous; photophobia. Eyelids feel as though they were made of parchment; conjunctiva injected. Total loss of strength. Nose, dark-red, erysipelatous, intense itching; soreness of nostrils. Mouth dry, putrid breath, much thirst. Stools yellowish, bloody, cadaverous. Urine hot, burning, frequent, scanty, involuntary at night. Scrotum and penis swollen; skin thick, dark-red, erysipelatous. Frequent erections, maddening, exquisite, voluptuous itching, which nearly sets him mad, better by gentle rubbing with a silk handkerchief, better from cool applications, worse from warmth and in bed. Great sleepiness, but cannot sleep on account of itching. Worse from sitting still, better from motion and in a cool place. Eruption on the arms a quarter of an inch thick, torn and ragged from violent scratching. More or less itching on other parts of the body, having no eruption.

After carefully looking up the case, the only remedy which I could see my way clear to prescribe was Rhus toxicondendron, which was given him in the CM "H S" potency, and allowed to act for forty-eight hours; at the end of that time I saw him and received the following report: Improved in every respect; swelling not so dark-red, and itching much less severe. Slept well the first night, the second night slept soundly. Sac. lac. was given, and the previous remedy allowed to act for two days more, with marked improvement in every respect. During this time the itching had disappeared, the swelling subsided and desquamation of the involved parts was taking place rapidly. In ten days from the beginning of treatment the whole difficulty was well except the torn and lacerated wrists, which were healed kindly during the next ten days.

Case II.—March 4th, 1885.—Thomas E. H., age, seventy-five; weight, one hundred and thirty pounds. Complains of feeling weak and lame all over, drawing in the knees, painful cracking of the knees when walking; darting pains from the hip to the hollow of the knee, aggravated when urinating; excessive backache, which causes him to lose much sleep early in the morning; must sit up or roll over in bed to get relief; brick-dust sediment in the urine, which adheres closely to the vessel. Constipation; ineffectual urging to stool, with some mucus discharge. The feet are cold and numb; cramp in calves at night, only relieved by standing on the floor. Urine passed with difficulty; painful pressure in the bladder before urinating; painful urging; urine passing in drops, with burning; *must stand with the knees bent and frequently change position* before the urine begins to pass. Don't feel that life is worth living, and has a notion to kill himself; he would do so if not afraid of the hereafter. Gave Nux vom.[cc]. Six doses were given, which in the course of six weeks, entirely relieved him of the entire train of symptoms.

Case III.—February 1st, 1882.—Mrs. L. W., married, thirty-five years of age, brown hair and eyes, full form, medium height, has had six children. Ever since she can remember has been subject to sick headaches. The pain begins all over the head, worse on the right side. Pain in temples and back of eyeballs, when pressing the eyes seems as though she could touch the pain; pressure does not aggravate the pain. Great sensitiveness of the eyes to light during the headache. Headache usually begins in the morning, growing worse during the day until she sleeps.

Headache always better after vomiting, vomiting difficult, aggravation during vomiting, relief after. Desire to remain perfectly quiet, noise or sudden jar causes intense agony, pains throbbing, beating with the pulse; worse from motion. Stooping causes a knife-like pain, mostly in right frontal eminence. Relief by binding the head up tightly, from hot water, worse from cold. Wants to lie with her head high. When raising the head feeling as though it would burst open, roaring and ringing in the ears all the time, soreness of the gums, canker-like (when nursing the baby), mostly in front, and on the inside of the lips. During the headache sense of a great load in the stomach, with goneness and oppression in the epigastrium. Frequent pains in the right hypochondrium and under the left shoulder-blade going through from the breast.

After the headache bruised feeling in abdomen; can hardly stand up. Constipation; stools causing sharp, hard pains just before the moving, relief after; during menses pain in lower part of abdomen, sharp, always worse at the beginning. A dull pain in sacrum. Rheumatic pains in both upper arms, worse when moving, worse in right side, disinclination to move. Feet are inclined to be cold, excruciating rheumatic pains in left hip, menstrual blood thick, dark, scanty; during menstruation face very red, fretfulness, cross, don't want people to move around. Gave Sanguinaria can.[cc] twelve powders.

February 25th.—On the 20th had a headache; began early in the morning, lasted all day and night, until the next day at noon; the first pains were as severe as usual, if not worse; the second day they grew much lighter, until they finally disappeared; other symptoms were present but not so severe; a new symptom of bright spots before the eyes, also clouds of gray, both of which passed off in two days. The remedy was allowed to act until March 8th. Has not had a headache for two weeks, although she has several times felt as though one was coming on.

July 16th.—Had a slight return of headache, which passed away in two hours; menses came Thursday without pain, and normal in quantity and color.

March 12th, 1886.—Remains perfectly well to this date, having had no return of the headache since the above record.

